# A stability-Indicating HPLC Method for Simultaneous Determination of Creatine Phosphate Sodium and its Related Substances in Pharmaceutical Formulation

**Published:** 2016

**Authors:** Zengkun Xie, Lihua Wei, Qin Yang, Min Yang, Hongchun Pan, Hong Liu

**Affiliations:** a *College of Pharmaceutical Sciences, Southwest University, Chongqing 400715, China. *; b *Chongqing Engineering Research Center for Pharmaceutical Process and Quality Control, Chongqing, China.*

**Keywords:** CPS, related substances, HPLC, stability-indicating method, method validation

## Abstract

The objective of the study was to develop a simple, specific and stability-indicating HPLC method for the simultaneous determination of creatine phosphate sodium (CPS) and its related substances in pharmaceutical formulation. Separation of creatine phosphate sodium from its major process impurities and degradation products was achieved on a Hypersil BDS C18 column (250 × 4.6 mm, 5 μm) with an aqueous mobile phase containing 0.2% (w/v) tetrabutylammonium hydroxide (TAH) and 0.2% (w/v) monopotassium phosphate adjusted to pH 6.6 with orthophosphoric acid at a flow rate of 1.0 mL min^-1^. The analytes were detected at 210 nm. Different chromatographic parameters were carefully optimized. The relative response factors for creatine, creatinine and creatinine phosphate disodium salt relative to CPS were determined. The method has been validated with respect to solution stability, system suitability, LOD, LOQ, linearity, accuracy, precision, specificity and robustness. The validation criteria were met in all cases. The developed method was successfully applied to determine the purity of CPS in pharmaceutical formulation.

## Introduction

**Figure 1:**
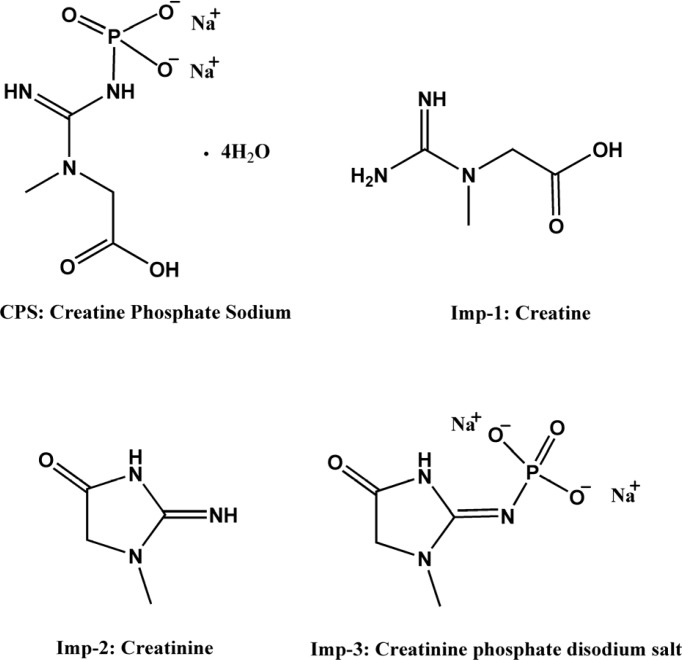
Chemical structures of CPS and its process related impurities

Creatine phosphate sodium (CPS), also called creatine phosphate disodium salt, is sodium salt of creatine phosphate (CP), which is an important biomolecule for energy storage and conversion in tissues (1). Clinically, it is frequently used as a myocardial protective agent for the treatment of diseases like myocardial ischemia, ventricular arrhythmias and myocardial infarction during heart surgical operation (2-4). CPS is soluble in water, but not in methanol, ethanol and acetonitrile, and can be decomposed into phosphate and creatine in the acidic solution because of the high-energy phosphate bond in molecular structure. This low fat solubility and instability in water make daily quality monitoring inconvenience. The chemical structure of CPS and its major process impurities (creatine, creatinine, creatinine phosphate disodium salt) are shown in Figure 1.

Some chromatographic methods have appeared in the literature for the quantification of creatine, creatine phosphate or creatinine in human urine, plasma, myocardial tissue and red blood cells, etc. (5-9). Several other methods have been published for the quantification of creatine phosphate and creatine in animal liver and brain (1, 10). Nevertheless, only a few methods have been reported for the analysis of CPS (11-12) or its related substances (13) in pharmaceutical formulation. After an extensive literature review and a series of experimental studies, it was found that the sample solution was unstable with the peak areas of the impurities increased significantly during the storage period; therefore it needs to be prepared freshly and stored in low temperature. The problem exists in the vast majority of the reported literatures, and it is inconvenient for routine quality testing in batch. Aside from this defect, the large standard deviation of peaks area may lead to the inaccuracy of the determination result as well as misjudgment. Moreover, an in-depth, systematic and comprehensive study for simultaneous determination of CPS and its related substances has not been reported, especially creatinine phosphate disodium salt is rarely reported. To sum up, none of the recognized pharmacopoeias or journals includes a relatively sound method for the simultaneous determination of CPS and related substances.

Taking the above-mentioned considerations into account, it is essential to develop a suitable stability-indicating HPLC method for the quantification of CPS, all the process impurities and the possible degradation products. How to improve the stability of solution is an important task in the present work. The newly developed method was validated in accordance with ICH guidelines (14). 

## Experimental

*Materials and reagents*

Reference standards of CPS, creatine and creatinine were obtained from National Institutes for Food and Drug Control (Beijing, China). The creatinine phosphate disodium salt reference standard was purchased from ChromaDex^TM^ (California, USA). CPS raw material and injections were kindly supplied by Hainan Quanxing Pharmaceutical. Acetonitrile (ACN) was chromatographic grade and procured from Spectrum Chemical Mfg. Corp. (Gardena, USA). Analytical grade orthophosphoric acid, sodium hydroxide, potassium permanganate and chromatographic grade KH_2_PO_4_ were purchased from Chengdu Kelong Chemical Reagent Company (Chengdu, China). Chromatographic grade tetrabutylammonium hydroxide (TAH) was purchased from Tianjin Guangfu Fine Chemical Research Institute. Demineralised water (≥ 18.0 Ω cm^-1^) was prepared with a Millipore Milli-Q plus purification system (Bedford, USA).

*Instruments*

A pH meter (Mettler Toledo, Germany) and a Mettler Toledo balance were employed in the preparation of sample solutions and mobile phase. Photo stability and thermal stability studies were carried out in Multi-Drug Stability Test Chamber (YSEI, China) and **DHG Series Electrothermal Constant-temperature Drying Box** (SANFA, China), respectively.

An Agilent 1260 Liquid Chromatograph (Agilent, USA) consisting of a quaternary pump, online degasser, column heater, autosampler and diode array detector was employed for analysis. Data collection and analysis were performed on an Agilent OpenLAB CDS ChemStation (Rev.C.01.03, Agilent Technologies). 

*Chromatographic conditions*

Compounds were separated on a Hypersil BDS-C18 column (250 mm × 4.6 mm I.D., 5 μm) (Dalian Elite Analytical Instruments Co., Ltd. Dalian, CHINA). The aqueous mobile phase contained 0.2% (w/v) TAH and 0.2% (w/v) monopotassium phosphate. The final pH of the mobile phase was adjusted to 6.6 by diluted orthophosphoric acid. The eluted compounds were monitored at 210 nm. Column temperature was set at 30 ˚C and the flow rate was 1.0 mL min^-1^. 20 μL of sample was injected to HPLC system. The mobile phase was filtered through 0.22 μm membrane filters and degassed prior to using.

*Preparation of* *solutions*

The aqueous diluent for standards and samples contained 0.2% (w/v) TAH and 0.2% (w/v) KH_2_PO_4_ (adjusted to pH 9.0 by caustic soda solution).

*Preparation of* *s* *tandard solution*

Stock solutions of the individual compounds (CPS, creatine, creatinine and creatinine phosphate disodium salt) were prepared by dissolving appropriate amount reference substance in the diluent. The mixed standard solution was prepared by diluting the respective stock solutions to the final concentrations in a 10 mL volumetric flask. The final concentration of CPS, creatinine phosphate disodium salt and other impurities were 100 μg mL^-1^, 2.5 μg mL^-1^, and 5 μg mL^-1^, respectively.

*Preparation of* *sample* *solution*

About 13.4 mg (water, 25%) of sample was accurately weighed and transferred to a 10 mL volumetric flask. A proper amount of diluent was added and shaken for 1 minute, then diluted up to the mark (1 mg mL^-1^). 1 mL of this solution was removed into a measuring flask of 10 mL. Then the flask was filled to full volume with the diluent to get the desired concentration (100 μg mL^-1^). The concentrated solution (1 mg mL^-1^) was for the analysis of creatine, creatinine and creatinine phosphate disodium salt, and the final solution was for the analysis of CPS.

*Stress* *study*

The stress conditions employed for the degradation study included base hydrolysis (1 N NaOH), acid hydrolysis (0.01 N H_3_PO_4_), thermal treatment (60 ˚C), oxidation (0.025 g mL^-1 ^KMnO_4_) and photolytic condition (4500 Lx ± 50). The samples were treated for 6 h, 40 minutes, 40 minutes for base, acid hydrolysis and oxidation, respectively. Whereas for thermal and photolytic condition studies, the samples were exposed for 10 days. After degradation studies, the stress samples were neutralized. Sample solutions were prepared as represented in “Preparation of sample solution”. The CPS peak purity of the stressed samples was monitored by the PDA detector in the wavelength range of 190-400 nm.

*Method development and optimization*

The chromatographic conditions including mobile phase composition, pH of mobile phase and diluent, detection wavelength were investigated to obtain the satisfactory analytical performance. 

*Selection of the mobile phase, detection wavelength and pH*

The most effective amount of TAH in the mobile phase was determined in five different concentrations (0.1, 0.2, 0.3, 0.4, 0.5%), and the most appropriate amount of KH_2_PO_4 _in mobile phase was 0.2%. The pH values of the mobile phase and the diluent were investigated in the range of 6.4-7.2 and 6.0-9.5, respectively. A suitable wavelength was required for the determination of CPS and three related substances simultaneously. The appropriate wavelength in the mobile phase was determined by wavelength scanning over the range of 190-400 nm.

*Method* *optimization*

The column was determined by comparing the three manufacturers of Agilent (Eclipse XDB-C18 4.6 × 250 mm, 5 μm), Elite (Hypersil BDS-C18 4.6 × 250 mm, 5 μm) and Waters (X Bridge^TM^ Shield RP18 4.6 × 250 mm, 5 μm). The flow rate was investigated at 0.8, 1.0 and 1.2 mL min^-1^. The column temperature and the sample volume were also investigated in the experiment.

*Method validation*

*System suitability*

System suitability checks are used for chromatographic methods to ensure that the system is sufficiently sensitive, specific and reproducible for the current analytical run (15). System suitability test was performed by assessing the six replicate injections of a system suitability solution. The system suitability parameters including retention time, number of theoretical plates, resolution, tailing factor, the peak area and retention time relative standard deviation (RSD, n = 6) of each compound were evaluated.

*Limit of quantitation* *(LOQ)* *and limit of detection* *(LOD)*

The LODs (S/N = 3) and LOQs (S/N = 10) for CPS, creatine, creatinine and creatinine phosphate disodium salt were estimated by injecting a series of dilute solutions with known concentrations, respectively. The precision study at the LOQ level was also carried out by injecting six individual preparations of the analytes standard solutions.

*Linearity*

The linearity of CPS and its related substances were studied by determining standard solutions at six different concentrations. The standard stock solution of CPS was diluted to 80-120% of the assay method concentration (100 µg mL^-1^). The linearity test solutions for creatine, creatinine, and creatinine phosphate disodium salt were prepared by diluting the impurity stock solutions to different concentrations, ranging from LOQ to 6 μg mL^-1^, and the range was approximately equivalent to 0.1-6.0% (w/w) of CPS in injections. All solutions were injected in triplicate.

*Relative response factor*

Relative response factor (RRF) was established for impurities 1, 2 and 3 as the ratio of slope of CPS and slope of impurities. Slope value of impurities obtained with linearity calibration plot (in the Table 2) was used for RRF determination. Slope value of CPS obtained with linearity calibration plot at impurities concentration was used for RRF determination. The linearity of CPS at impurities concentration was evaluated at six concentrations from LOQ concentration to 120% of 5 µg mL^-1^ (0.102-6.136 μg mL^-1^).

**Table 2.** Linearity, LOD and LOQ results for Creatine Phosphate sodium and related substances.

**Compd.**	**Regression equation**	**R** ^2^	**Linear range** **µg·mL**^−1^	**LOD ** **(** **ng** **)**	**LOQ ** **(** **ng** **)**
Imp-1	Y = 27.587 X+2.4935	0.9998	0.127～6.102	1.04	2.59
Imp-2	Y = 68.643 X+0.0293	0.9998	0.110～6.587	0.22	0.50
Imp-3	Y= 28.2999X+1.8199	0.9992	0.090～6.000	0.60	1.80
CPS	Y = 38.681 X-20.514	0.9995	83.021～118.305	0.90	2.00

*Accuracy*

Accuracy was determined by the method of standard addition recovery studies. This experiment was employed by adding the known amounts of CPS, creatine, creatinine, and creatinine phosphate disodium salt of three different concentrations into the drug samples whose concentrations were known. The different concentrations of the mixed samples were prepared in triplicate.

*Precision*

The repeatability of the proposed method was validated by analyzing the preparations of the six QC samples. The intermediate precision of the method was also verified on different days by different analyst, different columns and different HPLC instruments in the same laboratory. 

*Robustness*

Small and deliberate changes in HPLC parameters were made to determine the robustness of the analytical method. The HPLC parameters variation included the proportion of TAH or KH_2_PO_4_ (± 0.01%) in the mobile phase, pH (± 0.2 units) of the mobile phase, column temperature (± 5 ˚C), flow rate (± 0.2 mL min^-1^), wavelength (± 5 nm), and column batches. The selected parameters are those most likely to be changed and tend to have potential influence on the analytical results when the method is employed in practice (16). The drug samples were spiked with the impurity reference substances at impurity tolerance level. The effect of the HPLC parameters variation on the retention time and peak parameters were studied.

*Specificity*

The specificity of a method may be defined as the ability to accurately measure the analyte in the presence of all potential sample components (17). Assays were carried out for the stress samples against a qualified reference standard. The mass balance (% assay + % of impurities + % of degradation products) was calculated for all the samples.

*Solution stability*

The solution stability was assessed by analyzing the sample and standard solutions which were stored at room temperature for 12 h.

*Method application*

The validated HPLC method was successfully applied for the assay and related substances determination of CPS in drug substance and drug product. The drug products from three different manufacturers and the drug substance of three batches were included.

## Results and Discussion

*Method development and optimization*

*Selection of mobile phase*

**Figure 2:**
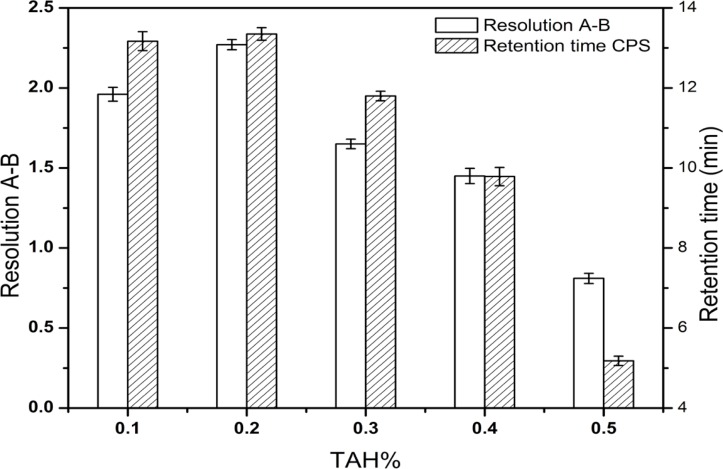
Effect of TAH% in mobile phase on the resolution between creatine (A) and creatinine (B) and the retention time of CPS. Data are represented as mean ± SD (n = 5).

**Figure 3:**
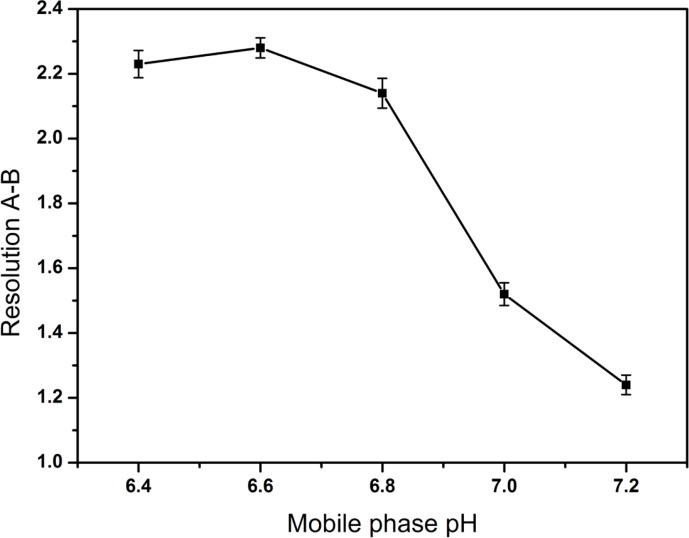
Effect of mobile phase pH on the resolution between creatine (A) and creatinine (B). Data are represented as mean ± SD (n = 5).

After a series of screening studies, the phosphate buffered solution containing the certain amount of TAH as an ion-pairing reagent was chosen as the mobile phase finally. TAH was added to the mobile phase to form nonpolar complexes with the analytes, which increased the retention time of ionic compounds. Furthermore, the addition of KH_2_PO_4 _was useful for improving peak shapes. In our preliminary study, the resolution between creatine and creatinine were found to be < 1.5 and it was considered as the critical parameter in further optimization. The molecular structures of CPS and the known related compounds (Figure 1) clearly implied that all compounds with amino group, phosphate group or carboxylic acid functional groups can be easily ionized. Therefore, the retention and separation of these compounds should be closely related to the pH and ionic strength of the mobile phase. Hence, the selection of mobile phase pH and the amounts of TAH and KH_2_PO_4_ in the mobile phase were crucial. Different quantities of TAH in the mobile phase were tested (Figure 2). The results showed that the most effective amount of TAH in the mobile phase was 0.2% (v/v), which was based on achieving reasonable resolution (R > 2), retention time and the good symmetry of the peaks. The retention time of CPS showed the dramatic changed with the variation of TAH amount. When the TAH concentration was increased or decreased from the optimum amount of 0.2% (v/v), it could shorten the analytical time, but it led to inadequate separation of creatine and creatinine (R < 2). The tailing factors were kept stable around 1.0-1.3, which indicated it had a good symmetry of the peaks. As shown in Figure 3, the best separation for the impurities was achieved when the pH value was 6.6.

*Improvement* *of* *solution stability*

**Figure 4:**
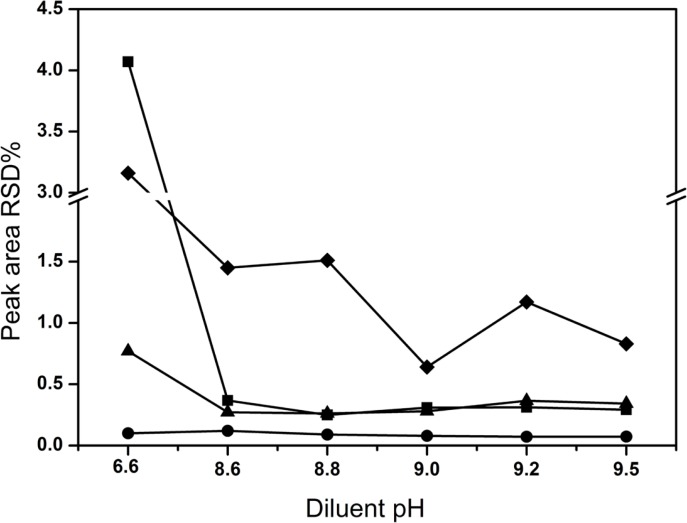
Effect of diluent pH on solution stability (■) creatine, (●) creatinine, (◆). creatinine phosphate disodium salt, (▲) CPS

The mobile phase was used as diluent in the preparation of sample solutions initially. For the sake of assessing the stability of solutions during analysis, the content of all compounds was determined after 6 h against freshly prepared standard solution at room temperature by HPLC. The RSD of peak areas for all analytes were calculated. However, the content of creatine and creatinine phosphate disodium salt significantly increased after 6 h compared with that of the freshly prepared. To counter this current situation of poor solution stability, active measures had been taken to search for a diluent that could assure the lower decomposition of CPS in the samples. In the former exploring research, it was observed that the diluent pH had important effects on CPS decomposition. The RSD of all analytes varied by changing the pH of the diluent in the range of 6.0-9.0 and the sample was relatively stable in the alkaline condition (Figure 4). Therefore, the pH of the diluent was further investigated over a range of 8.6-9.5. Finally, pH 9.0 was selected as the optimal diluent condition, although solvent peaks emerged. Obviously, the RSD values were the lowest (< 1.5%). The possible reason was as followed: CPS was typical alkaline (pH, 8.0-9.0), but in all the reported literatures the mobile phase was directly used as diluent which was of acidic, e.g. pH 3.0, 3.2, 3.3 and 7.0. In acidic condition, CPS was decomposed easily into creatine and creatinine phosphate disodium salt increasing the RSD value.

*Selection of the* *detection wavelength*

**Figure 5:**
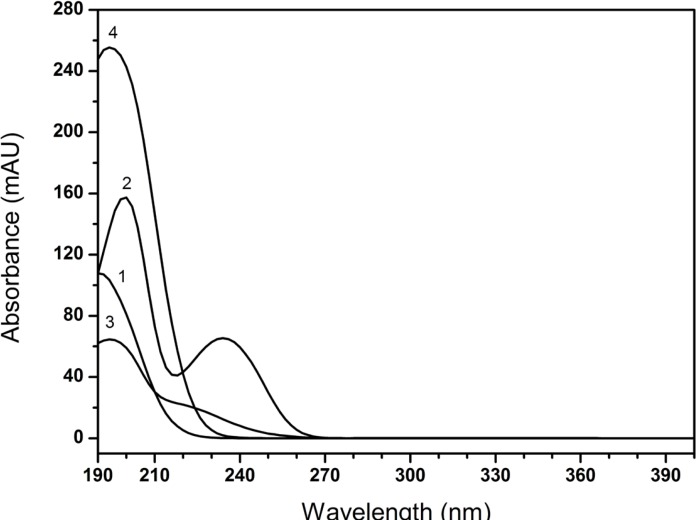
Overlay spectra of impurities and drug 1. creatine, 2. creatinine, 3. creatinine phosphate disodium salt, 4. CPS

**Figure 6:**
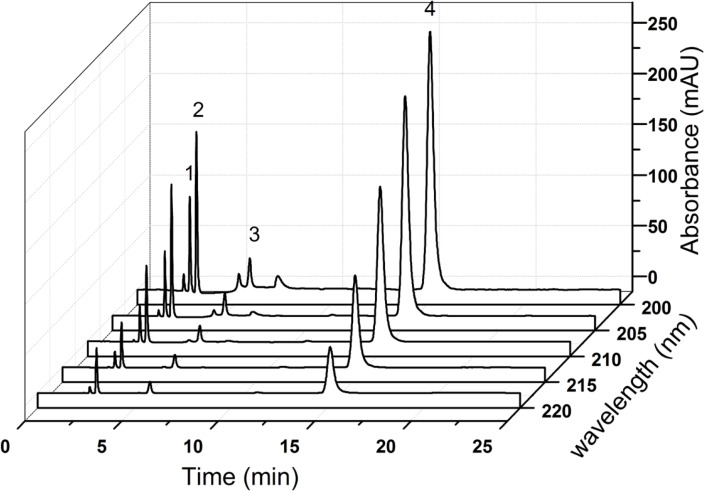
HPLC-DAD analysis of CPS and its impurities at different detection wavelengths 1. creatine, 2. creatinine, 3. creatinine phosphate disodium salt, 4.CPS

As shown in Figure 5, the maximum absorption wavelengths of all tested compounds were found in the range of 190-200 nm. Nevertheless, it was obviously noted that the baseline of the chromatogram was wandered at shorter wavelengths (< 210 nm, Figure 6) and it interfered with the determination of creatinine phosphate disodium salt. Though the diluent had a low detector response at 220 nm, the method was not sensitive enough to detect the degradation impurities at this wavelength (Figure 6). Therefore, the final detection wavelength was selected at 210 nm, since the drug had an optimal sensitivity and better resolution at this wavelength. Low quantities of impurities can be detected accurately, which showed acceptably accorded with the literature (3).

*Further optimization of the method*

**Figure 7:**
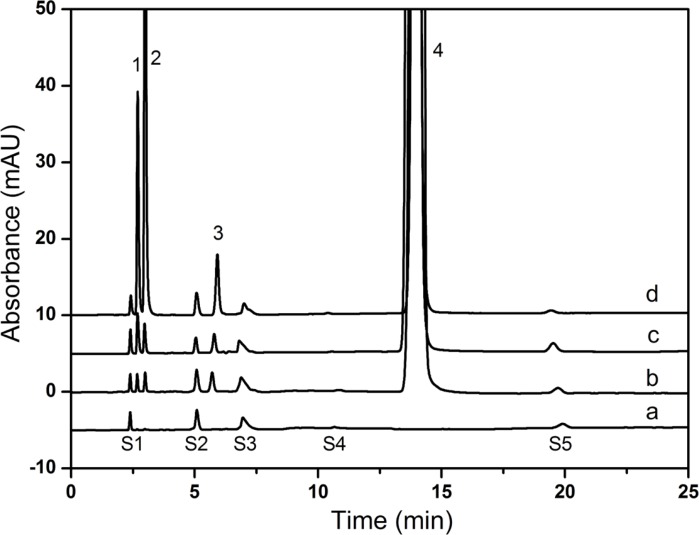
Typical chromatograms of (a) blank diluent, (b) CPS drug substance, (c) CPS for injection, (d) Mixing standard solution 1. creatine, 2. creatinine, 3. creatinine phosphate disodium salt, 4. CPS, S1-S4: solvent peak

After an extensive study, the flow rate was selected at 1.0 mL min^-1 ^finally. The chromatographic separation was achieved on a Hypersil BDS-C18 column (250 mm × 4.6 mm, 5 μm) maintained at 30 ˚C. The typical HPLC chromatograms (Figure 7) represented satisfactory separation of all compounds. 

*Identification of chromatographic peaks*

As shown in Figure 7, all components were identified as 1, creatine **2**, creatinine **3**, creatinine phosphate disodium salt **4**, CPS. Identification of the peaks in the sample solutions (Figure 7b and 7c) was performed by comparing their relative retention times with those obtained in the standard chromatograms (Figure 7d). Five solvent peaks which were observed in the blank diluent (Figure 7a) could be separated from all the studied compounds successfully. The relative retention times of solvent peaks were about 0.16, 0.35, 0.52, 0.72 and 1.37, respectively. The retention times for creatine, creatinine, creatinine phosphate disodium salt and CPS were about 2.7, 3.0, 5.9 and 14.0 min, respectively. Because of the poor reproducibility of ion-pair chromatography (18), the retention time of CPS shifted in the range of 14.0-15.0 min.

*Method validation*

*System suitability*

RSD (n = 6) of peak area for all compounds was found less than 1.0% and the RSD of retention time was found less than 0.2% in this study. In addition, the USP tailing factors were less than 1.5 and the resolution between two adjacent peaks (creatine and creatinine) were greater than 2.2. As shown in Table 1, all the values were in accordance with the acceptability criteria.

**Table 1:** System suitability results of the proposed method

**Compd.**	**t**_R_ **(min)**	**RRT**	**N**	**R**	**T**	**RSD** a	**RSD** b
Imp-1	2.699	0.19	7208	2.21	1.25225	0.05	0.81
Imp-2	2.997	0.21	7497	2.28	1.28082	0.05	0.26
Imp-3	5.923	0.42	10818	3.73	1.08252	0.07	0.48
CPS	14.070	1.00	14269	8.70	1.01630	0.12	0.12

t_R_: Retention time, RRT: Relative retention time, N: Theoretical plate number, R: Resolution, T: tailing factor, RSD

a : RSD % of retention time, RSD

b : RSD % of peak area.

*Limit of quantitation* *(LOQ)* *and limit of detection* *(LOD)*

 As shown in Table 2, the LOQs of CPS, creatine, creatinine and creatinine phosphate disodium salt (sample size, 20 µL) corresponded to 0.1%, 0.013%, 0.0025%, and 0.009% of the target sample preparation concentration, respectively. The precisions at the LOQ concentration were entirely below 2%. These results suggested that the proposed method was highly sensitive and accurate in determining trace impurities. 

*Linearity*

As shown in Table 2, the standard curves were constructed by the least square regression method. The results showed that peak area and concentration appeared a good linear relationship within the range of concentrations indicated above.

*Relative response factors*

The calibration curve of CPS at impurities concentration was Y = 36.808 X + 1.0315, R^2^ = 1.0. Established RRF value for creatine, creatinine, and creatinine phosphate disodium salt were 1.335, 0.536 and 1.301, respectively. These results demonstrated that the UV responses for creatine and creatinine phosphate disodium salt were lower than the active (CPS). However, compared to creatine and creatinine, creatinine phosphate disodium salt has higher UV response. Because the relative response factors were all out of the range of 0.9-1.1, the main component self-compare with calibration factor method for the determination of the related substances was used when there was no impurity reference substance.

*Accuracy*

 The results are presented in Table 3 and it revealed that the recovery of CPS injection formulation ranged from 99.05 to 101.90% and the RSD (n = 9) was 1.34%. The recoveries of the three impurities were in the range of 100.58-104.48% and the RSD (n = 9) were not greater than 2.0%. In conclusion, the proposed method was highly accurate for the quantitative analysis.

**Table 3:** Accuracy results for different levels of CPS and related substance in sample solutions

**Added** **level** **% (n=3)**	**Assay determination**			**Added** **level%** **(n=3)**	**Related substance determination**						
	**Added** **(μg** ** mL** ^-1^ **)**	**Recovery** **%**	**RSD** **%**		**Added** **(μg** ** mL** ^-1^ **)**	**Imp-1**		**Imp-2**		**Imp-3**	
						**Recovery%**	**RSD%**	**Recovery%**	**RSD%**	**Recovery%**	**RSD%**
80	83.98	101.90	1.34	50	2.5	104.48	1.56	98.21	0.77	99.26	2.00
100	102.07	99.07		100	5.0	102.31		98.92		96.56	
120	122.45	99.05		150	7.5	100.58		97.29		98.61	

*Precision*

In repeatability study, the RSD (n = 6) of the assay method was 0.32% and the values of related substances method ranged from 2.77 to 13.2%, which were within the acceptable limit of 2.0% and 15%, respectively. In intermediate precision study, the RSD (n = 12) for assay was 0.59 % and the RSD for all the related substances determination were in the range of 5.33-13.01%, while the acceptance criteria were below 2.0% and 20%, respectively. The developed method showed a good precision through the entire data.

*Robustnes* *s*

In most of the experimental conditions, the alterations of HPLC parameters didn’t significantly affect the system suitability requirements (the relative retention times, tailing factor, resolution, etc.). The content variability of CPS and the impurities were all within ± 2% (RSD, n = 6). Variations of detection wavelength had a certain effect on the compounds absorption intensities (peak responses). However, the solvent peak (S2) and creatinine phosphate disodium salt were closely eluting when the proportion of TAH decreased. Therefore, it was concluded that the method was sensitive to the detection wavelength and TAH% in mobile phase. 

*Specificity*

**Figure 8:**
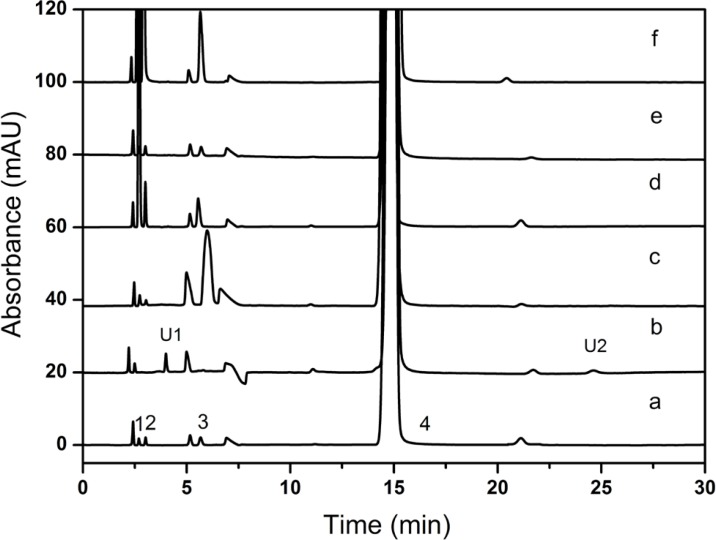
Overlay chromatograms of CPS degradation study (a) unbroken sample, (b) peroxide stress, (c) base stress, (d) acid stress, (e) photolytic stress, (f) heat stress U1: unknown impurity (RRT = 0.78), U2: unknown impurity (RRT = 1.32

6% H_2_O_2_ was performed in the oxidation study and 1N HCl was carried out in the acid hydrolysis. However, HCl had a negative peak which may interfere with the determination of creatinine phosphate disodium salt and similar problems still existed in the usage of H_2_O_2_. For the above reasons, H_3_PO_4_ and KMnO_4_ were chosen as replacements. All the results of the forced degradation studies were presented in Table 4. Representative chromatograms obtained under all stress conditions were shown in Figure 8.

**Table 4:** Results of forced degradation studies of CPS injection formulation

**Stress conditions**	**CPS %**	**Total impurities (%)**	**Mass balance (%)**	**Remarks**
Control	101.15	0.09	101.24	—
Base hydrolysis (1 N NaoH, 6 hours)	97.48	3.31	100.79	Imp-3 was major degradation product.
Acid hydrolysis (0.01 N H_3_PO_4_, 40 mins)	100.09	1.26	101.35	Imp-1, 2, 3 increased.
Thermal treatment (60 ℃, 10 days)	95.58	6.55	102.13	Imp-1, 2, 3 increased.
Oxidation condition (0.025 g mL^-1^ KMnO_4, _40 mins)	100.38	0.13	100.51	Two unknown degradation products formed designated as U1, U2. Imp2 and imp-3 were undetected.
Photolytic condition (4500 Lx ± 500, 10 days)	100.81	0.10	100.91	Mild degradation was observed.

The CPS samples were not stable and easily degraded under the acid, base, hydrogen peroxide or thermal conditions, and no dramatically degradation was observed when exposed to photolytic condition. The mass balance of the stressed samples was in the range of 100.51-102.13%, and the stability-indicating power of the method was proved. The peak purity was found to be greater than 0.9998, which indicated that new degradation products that could co-elute with CPS were undetected. The figure also showed that baseline resolution (R > 1.5) was achieved for all investigated compounds. During the oxidative degradation process, two unknown degradation products were observed at 4.204 min (U1, RRT = 0.28) and 24.782 min (U2, RRT = 1. 3), respectively. Creatinine and creatinine phosphate disodium salt were undetected, and those may be degraded. The specificity test results proved that all the potential impurities of the CPS samples could be separated completely and determined accurately. 

*Solution stability*

The results showed that the peak areas of CPS and its impurities remained almost unchanged and no significant degradation was observed during this period. So the results suggested that these solutions could remain stable in a sufficient time to complete the analytical process.

*Method application*

As shown in Table 5 and Table 6, the mean percentage of CPS and the three related substances were in good agreement with the label claimed. Furthermore, the *RSD* values were all consistent with the ICH impurity guidelines. The results clearly demonstrated that all compounds were determined with on other impurities interference, and the developed HPLC method was well applied for routine analysis of CPS in pharmaceutical formulation.

**Table 5:** Determination of CPS and its related substances in injection formulation

**Sample**	**Known related substances (%)**			**Unknown** **Impurity (%)**	**Total Impurities (%** **)**	**Assay (%** **)**
	**Imp-1**	**Imp-2**	**Imp-3**			
Brand I	0.05	0.01	0.10	ND	0.14	101.44
Brand II	0.05	0.02	0.03	ND	0.09	103.28
Brand III	0.03	0.01	0.07	ND	0.10	102.38

ND: not detected.

**Table 6:** Determination of CPS and its related substances in drug substance

**Sample**	**Known related substances (%)**			**Unknown** **Impurity (%)**	**Total Impurities (%** **)**	**Assay (%** **)**
	**Imp-1**	**Imp-2**	**Imp-3**			
Batch I	0.03	0.01	0.02	ND	0.05	100.31
Batch II	0.03	0.01	0.01	ND	0.05	100.36
Batch III	0.03	0.00	0.02	ND	0.04	100.99

ND: not detected

## Conclusions

In this study, a new HPLC method was established for the quantitative determination of CPS in the presence of its three process-related impurities and other potential degradation products. Compared with all the reported methods (11-13), the proposed method was remarkably improved in the solution stability and resolution. The proposed method was also proved to be simple, sensitive, linear, precise, accurate, robust and specific. Besides, the present analytical work is the first, in-depth, systemic and overall research about CPS and the related substances. The newly established method has solved the common problem in quality control during the pharmaceutical production and storage process. Thus, the developed method can be recommended for routine analysis and stability studies of CPS in the relevant forms.
